# Psychometric properties of the Greek TCI-R and its clinical correlates: schizotypy and the self-regulation of affective and cognitive functioning

**DOI:** 10.7717/peerj.1830

**Published:** 2016-03-22

**Authors:** Stella G. Giakoumaki, Leda Karagiannopoulou, Sándor Rózsa, Chrysoula Zouraraki, Penny Karamaouna, C. Robert Cloninger

**Affiliations:** 1 Department of Psychology, University of Crete, Rethymno, Greece; 2 Department of Psychiatry, Washington University School of Medicine, St. Louis, Missouri, USA

**Keywords:** Temperament, Character, Resilience, Schizotypy, Affect balance, Cognition, Temperament character inventory-revised

## Abstract

**Background.** The revised Temperament and Character Inventory (TCI-R) measures Cloninger’s psychobiological model of personality. The average effects of individual temperament and character traits have been associated with schizotypy and with impaired regulation of affect and cognition. We extended prior research by testing predictions about the association of specific multidimensional configurations of temperament and character traits on schizotypy, affect balance, and self-perceived cognitive functioning.

**Method.** A well-educated sample of native Greeks (*N* = 483), completed a new Greek translation of the TCI-R, as well as the Schizotypal Personality Questionnaire (SPQ), the Positive/Negative Affect Schedule (PANAS) and the Cognitive Failures Questionnaire (CFQ). The factor structure of the TCI-R was examined with exploratory and confirmatory tests. Associations between reported measures were examined with correlational and regression analyses.

**Results.** The TCI-R had good psychometric properties as expected from studies in other countries. As predicted, specific configurations of temperament and character were associated with schizotypy, negative affect balance, and cognitive lapses. The “Borderline/Explosive temperament” (high Novelty Seeking, high Harm Avoidance, low Reward Dependence), “Schizotypal/Disorganized character” (low Self-directedness, low Cooperativeness, high Self-transcendence), and “Low Ego Strength/Fragile” profile (high Harm Avoidance, low Persistence, low Self-Directedness) were each strongly associated with higher stereotypy, negative affect balance (low positive affect and high negative affect), and subjective cognitive lapses compared to their contrast groups.

**Discussion.** Multidimensional TCI profiles are strongly related to individual differences in schizotypy and self-reported regulation of affect and cognition. The Greek translation of the TCI-R is psychometrically sound and useful for clinical assessment and research.

## Introduction

Cloninger’s biopsychological model differentiates between Temperament and Character dimensions of personality (Cloninger, Svrakic & Przybeck, 1993). *Temperament* refers to individual differences in percept-based habits and skills that are regulated by the limbic system (Cloninger, 1994) and measured by four independently inherited dimensions that are moderately stable throughout life (Cloninger & Svrakic, 1997): *Novelty Seeking* (NS) refers to a tendency towards exploratory activities in response to novelty and is hypothesized to be mediated by a dopaminergic behavioural activation system; *Harm Avoidance* (HA) refers to pessimistic worrying in anticipation of problems that is hypothesized to be mediated by a serotonergic behavioural inhibition system; *Reward Dependence* (RD) is defined as a tendency to maintain behaviours in response to reward by others and is mediated by a noradrenergic behavioural maintenance subsystem; *Persistence* (PS) is an independent dimension and refers to a tendency to perseverance despite frustration and fatigue. In contrast, *Character* refers to individual differences in concept-based goals and values that involve semantic and autobiographical learning (Cloninger, 1994) and is measured by three dimensions that mature in a stepwise fashion from infancy through adulthood (Cloninger & Svrakic, 1997): *Self-Directedness* (SD) is defined as the ability for self-determination and will-power; *Cooperativeness* (CO) refers to the capacity for empathy and compassion for others; and *Self-Transcendence* (ST) is related to individual differences in transpersonal experience and spirituality.

Three sets of complex interactions among these traits have been described and can be depicted as a linked network of three cubes, as described by Cloninger (for review see TCI Research Training Course at psychobiology.wustl.edu). The temperament cube involves interactions among NS, HA, and RD, whereas the character cube involves interactions among SD, CO, and ST (Cloninger, 2004). These two cubes are in turn linked by the interactions of PS with HA from the temperament cube and SD from the character cube (Cloninger et al., 2012). These interactions among HA, PS, and SD are referred to as the resilience cube, since these three dimensions are strongly related to resilience and well-being (Cloninger et al., 2012; Eley et al., 2013). These configurations of variables in individual people are associated with distinctive patterns of functioning and so contain more information than sums of the average effects of their constituents (Cloninger et al., 2012).

Cloninger’s model was initially tested with the Tridimensional Personality Questionnaire (TPQ), a 100-item questionnaire measuring NS, HA and RD (Cloninger, 1987). As the initial model was revised and supplemented with the three character dimensions, TPQ evolved into the Temperament and Character Inventory (TCI), a dichotomous 240-item questionnaire comprising all seven dimensions (Cloninger et al., 1994). The Revised Temperament and Character Inventory (TCI-R; Cloninger, 1999; Goncalves & Cloninger, 2010) is the product of the second revision of the initial instrument. Its main modifications are (a) the conversion of the response format into a five-point Likert-scale (ranging from 1: definitely false to 5: definitely true), (b) the further categorisation of the higher-order temperament and character dimensions into more detailed sub-dimensions (16 for temperament and 13 for character; Table 1) and (c) the addition of five validity items. TCI-R is a widely used instrument for the assessment of personality that has been adapted and validated in over twenty countries (e.g., Pelissolo et al., 2005 in France; Martinotti et al., 2008 in Italy; Dzamonja-Ignjatovic et al., 2010 in Serbia; Snopek et al., 2012 in Czech Republic) with high coefficients of internal consistency (e.g., Fresán et al., 2011; Goncalves & Cloninger, 2010; Tilov et al., 2012), test-retest reliability (e.g., Hansenne, Delhez & Cloninger, 2005; Martinotti et al., 2008; Pelissolo et al., 2005), construct and predictive validity for personality disorders (e.g., Dzamonja-Ignjatovic et al., 2010; Fossati et al., 2007; Martinotti et al., 2008).

**Table 1:** Higher-order scales and subscales of the TCI-R.

	Subscales
**Novelty Seeking (NS)**	Exploratory excitability vs stoic rigidity
	Impulsiveness vs Reflection
	Extravagance vs Reserve
	Disorderliness vs Regimentation
**Harm Avoidance (HA)**	Anticipatory worry & Pessimism vs Uninhibited optimism
	Fear of uncertainty
	Shyness with strangers
	Fatigability & asthenia
**Reward Dependence (RD)**	Sentimentality
	Openness to warm communication vs aloofness
	Attachment
	Dependence
**Persistence (PS)**	Eagerness of effort vs laziness
	Work hardened vs spoiled
	Ambitious vs underachieving
	Perfectionist vs Pragmatist
**Self-Directedness (SD)**	Responsibility vs Blaming
	Purposefulness vs lack of goal-direction
	Resourcefulness
	Self-acceptance vs Self-Striving
	Enlightened second nature
**Cooperativeness (CO)**	Social Acceptance vs Social intolerance
	Empathy vs Social disinterest
	Helpfulness vs unhelpfulness
	Compassion vs Revengefulness
	Pure-hearted Conscience vs Self-serving Advantage
**Self-Transcendence (ST)**	Self-forgetful vs Self-conscious Experience
	Transpersonal Identification vs Self-Differentiation
	Spiritual Acceptance vs Rational Materialism

Interestingly, a configuration of Temperament and Character traits, namely increased HA and ST along with low SD and CO, has been associated with schizotypy (Bora & Veznedaroglu, 2007; Daneluzzo, Stratta & Rossi, 2005; Smith et al., 2008), a personality structure referring to “liability” to schizophrenia and spectrum disorders (Lenzenweger & Korfine, 1995). Additionally, Temperament and Character traits have been proposed as endophenotypic markers of severe psychiatric disorders such as schizophrenia (Bora & Veznedaroglu, 2007; Nitzburg, Malhotra & Derosse, 2014; Sim et al., 2012; Smith et al., 2008; Song et al., 2013), bipolar disorder (Almeida et al., 2011; Greenwood et al., 2013) and depression (Farmer et al., 2003; Teraishi et al., 2014) (for review see Cloninger, Zohar & Cloninger, 2010). Rather than looking only at the average association of individual TCI traits with schizotypy, as has been done in prior studies, we sought to test the effect of multidimensional profiles. For example, we hypothesized that particular contrasts within each cube would be strongly associated with schizotypy. Specifically, based on our understanding of the constituents of schizotypy and TCI profiles, we extrapolated from prior literature to predict that schizotypy would be strongly associated with the explosive profile (i.e., high HA, high NS, and low RD) in the temperament cube because this profile is typical of people with borderline personality disorders and brief reactive psychoses (Cloninger, 2004). Likewise, we predicted that the disorganized or schizotypal profile (i.e., low SD, low CO, and high ST) in the character cube would be associated with schizotypy because this is typical of people with schizotypal personality disorders and relatives of people with schizophrenia (Cloninger, 2004; Smith et al., 2008). Finally, we predicted the fragile profile (i.e., high HA, low PS, and low SD) in the resilience cube would be associated with schizotypy because low resilience is typical of people with vulnerability to schizophrenia and low ego strength, which involves being neurotic (i.e., high HA, low SD) and easily discouraged (i.e., low PS) (Smith et al., 2008; Nettle, 2006). Although borderline, disorganized, and fragile configurations often overlap, they do occur separately. We predicted that each would be associated with schizotypy, either individually or in combination.

Temperament and Character traits have also been associated with impaired performance in cognitive tasks in clinical samples, subjects at high risk for schizophrenia, and in the general population. For example, in clinical samples, working memory and problem solving are positively associated with SD and problem solving is also negatively associated with ST in schizophrenia patients (Boeker et al., 2006), implicit learning is negatively associated with RD (Galderisi et al., 2011), and perseveration is positively associated with ST (Pignatti & Bernasconi, 2013) in bulimia nervosa patients. Likewise in psychiatric high-risk groups, working memory and crystallised IQ are positively associated with SD and CO in non-psychotic siblings of schizophrenia patients, but not in the general population (Smith et al., 2008). In non-clinical populations, cognitive flexibility is positively associated with HA but is negatively associated with RD (Guillem et al., 2008), and verbal memory is negatively associated with HA but is positively associated with high SD and CO (Hori et al., 2012).

In addition to heritable influences, personality traits are regulated by cultural factors (Allik & McCrae, 2004). According to the Universal Declaration on Cultural Diversity of UNESCO, *“culture should be regarded as the set of distinctive spiritual, material, intellectual and emotional features of society or a social group, and that it encompasses, in addition to art and literature, lifestyles, ways of living together, value systems, traditions and beliefs”* (http://portal.unesco.org/en/ev.php-URL_ID=13179& URL_DO=DO_TOPIC& URL_SECTION=201.html). Although the cross-cultural applicability of TCI-R has been well-documented in several countries, there is no current study examining the properties of the instrument in Greece during the “full-blown” economic crisis, which has probably affected all the afore-mentioned characteristics of the Greek population. Therefore, the aim of the present study was three-fold: (a) to explore the factor structure and external validity of the TCI-R, (b) to describe the internal consistency, gender differences, and correlations among the TCI-R dimensions and (c) to examine the association of TCI-R measures with schizotypy, affect balance, and self-perceived cognitive functioning in a well-educated Greek community sample.

## Materials and Methods

### Participants

Five-hundred and twenty adults anonymously completed the TCI-R (Cloninger, 1999), the Schizotypal Personality Questionnaire (SPQ; Raine, 1991), the Positive and Negative Affect Schedule (PANAS; Watson, Clark & Tellegen, 1988) and a form assessing their medical history. Thirty-seven of those returned invalid TCI-R inventories (i.e., they responded incorrectly in any of the five validity items); as the present study focused on the psychometric properties of TCI-R and on associations between its dimensions with other measures, we excluded these participants from the analyses. Therefore, the final sample consisted of 483 community participants (62.90% females; age-range: 18–65 years; age mean ± SD: 34.77 ± 10.90) with valid TCI-R and SPQ inventories; of those, 480 participants also returned valid PANAS. A sub-sample consisting of 352 participants also completed the Cognitive Failures Questionnaire (CFQ; Broadbent et al., 1982). The majority of the participants (42.65%) had a university degree, 33.95% had completed postgraduate studies and 23.40% had a high-school degree. Exclusion criteria were personal history of head trauma, medical or neurological conditions, current use of prescribed or recreational drugs and personal or family history of DSM-IV disorders. The present project was part of the Prefrontally-Mediated Endophenotypes in the Schizophrenia spectrum (PreMES) study. The study was approved by the Research Ethics Committee of the Department of Psychology in the University of Crete (approval number: 36/12-09-2012), the central Research Ethics Committee of the University of Crete (approval number: 06/ 18-10-2012) and the Bureau for the Protection of Personal Data of the Greek state (approval number: ΓN∕EΞ/749-1/21-12-2011). Following presentation of the study’s methods, all participants received a detailed information sheet and gave informed consent before participation.

### Assessment of personality, positive/negative affectivity and self-perceived cognitive functioning

*Revised Temperament and Character Inventory* (TCI-R; Cloninger, 1999). TCI-R is a 240-item self-report questionnaire assessing the four temperament (Harm Avoidance, Novelty Seeking, Reward Dependence and Persistence) and three character higher-order dimensions (Self-Directedness, Cooperativeness and Self-Transcendence). Each higher-order dimension if further divided into sub-scales. Items are rated in a five-point Likert scale, with responses ranging from definitely false to definitely true. Inaccurate and hasty responding is measured by five validation items. Validation items do not ask whether the respondent agrees/disagrees with a statement but require that he/she gives a pre-specified response (e.g., “Please circle the number four, this is a validity item”); they are used to confirm that the respondent understands what he/she has to do and continues to pay attention to the task at hand. The scores for the subscales are the sum of specified items and the scores for the higher-order dimensions are the sum of the respective subscales. The TCI-R translation in Greek was performed by S. G. Giakoumaki and was back-translated by a bilingual translator, who was blind to the original version of the instrument. The back-translation was reviewed and approved for psychometric testing in this validation project by C. R. Cloninger.

*Schizotypal Personality Questionnaire* (SPQ; Raine, 1991; Tsaousis et al., 2015): SPQ was administered for the assessment of schizotypy. SPQ is a 74-dichotomous item (yes/no) questionnaire organized into nine subscales that mirror the DSM diagnostic criteria of schizotypal personality disorder, including ideas of reference, excessive social anxiety, odd beliefs, unusual perceptual experiences, odd behaviour, no close friends, odd speech, constricted affect, and suspiciousness. Separate positive schizotypy (i.e., Cognitive-perceptual factor including odd beliefs, ideas of reference, unusual perceptual experiences, suspiciousness scores), negative schizotypy (i.e., Interpersonal factor consisting of social anxiety, no close friends, emotional blunting, suspiciousness scores), and Disorganization (odd speech, odd behaviour scores) factors (Raine et al., 1994), as well as a total score, are computed. Cronbach’s alphas for the SPQ scores in the present study were: 0.77 for the Cognitive-Perceptual factor, 0.76 for the Interpersonal factor, and 0.50 for the Disorganization factor.

*Positive and Negative Affect Schedule* (PANAS; Sideridis et al., 2006; Watson, Clark & Tellegen, 1988): PANAS was used for the assessment of positive/negative affectivity. It is a 20-item self-report scale (10 items assessing positive affect and 10 items assessing negative affect) designed to provide measures of positive and negative affect. Items are rated on a 5-point Likert scale ranging from 1 (very slightly or not at all) to 5 (extremely) during the past week. The Positive Affect sub-scale is derived with the sum of the scores in the ten respective items and higher scores indicate higher levels of positive affect. The Negative Affect sub-scale is derived with the sum of the scores in the ten respective items and higher scores indicate higher levels of negative affect. Cronbach’s alphas in the present study were: 0.74 for the Positive Affect sub-scale and 0.86 for the Negative Affect sub-scale.

*Cognitive Failures Questionnaire* (CFQ; Broadbent et al., 1982): CFQ is a 25-item self-report questionnaire measuring the frequency of cognitive failures in everyday life. It is scored on a five-point Likert scale, ranging from 0 (never) to 4 (very often). It is scored as the sum of the responses with high scores indicating increased propensity to cognitive failures. Wallace, Kass & Stanny (2002) found that the CFQ consists of four principal factors, namely distractibility, memory, blunders, and memory for names, which were further confirmed with confirmatory factor analysis (Wallace, 2004). These additional factors were also included in the present study. Cronbach’s alphas for the CFQ scores in the present study were: 0.78 for memory, 0.82 for distractibility, 0.66 for blunders, and 0.80 for memory for names.

### Statistical analyses

The factor structure of the TCI-R scales was examined with principal component analyses using the Promax rotation method separately for the temperament and character subscales; components with Eigenvalues >1 were accepted. We chose to run separate analyses for the temperament and character subscales due to the lack of linear relations between these personality domains (Cloninger, 2000). The temperament and character factor structures of the Greek TCI-R were compared with the original version of the TCI-R (validated in St. Louis, MO, USA with 962 subjects). Orthogonal Procrustes rotations (Schönemann, 1966; Schumacker & Beyerlein, 2000) and congruence coefficients between the factorial matrices were estimated to demonstrate the equivalence between the American and Greek TCI-R. We used maximum likelihood confirmatory factor analyses (CFA ran with AMOS Arbuckle, 2006) over the covariance matrices of the temperament and character subscales to test factor structures. The following goodness-of-fit indices were estimated: *χ*2 statistic, comparative fit index (CFI), goodness-of-fit index (GFI), root mean square error of approximation (RMSEA) and standardized root-mean-square residual (SRMR). CFI and GFI values >0.90 indicate an acceptable fit (Byrne, 2001; Kline, 1998). For the RMSEA, a cut-off value ranging from 0.05 or lower indicates good model fit and values up to 0.08 represent moderate fit. An SRMR of between 0 and 0.05 indicates a good fit, and between 0.05 and 0.10, an acceptable fit (Hu & Bentler, 1999; Schermelleh-Engel, Moosbrugger & Müller, 2003). Means, standard deviations and Cronbach’s alpha coefficients were estimated both for the scales and subscales. Gender differences were examined with univariate analyses of variance, with TCI-R scores as the dependent variables and gender as a grouping factor. Pearson’s correlations were conducted among the TCI-R higher-order dimensions and between TCI-R higher-order dimensions and age, PANAS, SPQ and CFQ measures. Associations of the TCI-R scales and subscales with SPQ total score were examined with a series of stepwise regression analyses; in these analyses, the dependent variable was the SPQ total score and the predictors in the respective models were (a) the Temperament scales, (b) the Character scales, (c) both Temperament and Character scales and (d) Temperament and Character subscales. We also formed personality profiles according to the three personality “cubes” by dividing our sample into participants scoring above or below the median for each Temperament and Character dimension, as recommended and validated by Cloninger and his colleagues for accounting for complex dynamics of personality interactions (Cloninger, Svrakic & Svrakic, 1997; Cloninger et al., 2012). Group-wise comparisons in the PANAS, SPQ and CFQ scores were performed with univariate analyses of variance.

## Results

### Factor structure of the TCI-R scales

*Principal Component analyses*: For the temperament subscales, four factors with Eigenvalues >1 were extracted, which accounted for 66.61% of the variance: Factor 1: PS (explained variance: 29.04%), Factor 2: HA (explained variance: 16.20%), Factor 3: RD (explained variance 14.44%) and Factor 4: NS (explained variance: 6.92%). Subscale scores loaded on the expected factors with loadings >0.40. However, the subscale NS1 loaded weakly on the NS factor (0.21) and also loaded moderately on the HA and RD factors (−0.39 and 0.42, respectively). The subscale RD1 also loaded positively the HA factor (0.58) and RD4 also loaded negatively the NS factor (−0.49). For the character subscales, the three expected factors with Eigenvalues >1 were revealed, which accounted for 59.29% of the variance: Factor 1: CO (explained variance: 29.09%), Factor 2: SD (explained variance: 19.52%) and Factor 3: ST (explained variance: 10.68%). As with the temperament subscales, scores loaded on the expected factors with loadings >0.40. However, the subscale SD4 loaded weakly on the SD factor (−0.08) and also loaded on the CO and ST factors (0.65 and −0.40, respectively).

*Congruence and Procrustes-rotated structure:* We used orthogonal Procrustes rotation in order to examine the cross-cultural replicability of the TCI-R factor structure. All subscales had the highest loading on the expected factor, except NS1 and SD4. RD1 and RD4 also had large secondary loadings. Out of the 29 subscales, a significant congruence coefficient at *p* < 0.01 (congruence values ≥99%) was revealed for 27 facets, and another two (NS1 and CO2) had a significant congruence coefficient at *p* < 0.05 (congruence values ≥95%). A congruence coefficient ≥0.90 or higher is considered as strong evidence of factor replication (Barrett, 1986). There were no differences in any subscales between the Greek and the American normative sample matrix. A detailed description of the findings is presented in Table 2.

**Table 2:** Orthogonal Procrustes rotated structure with congruence coefficients for the TCI-R subscales. Loadings with absolute values ≥0.40 are shown in bold. Expected loadings according to Cloninger’s theoretical framework are marked in gray background.

Temperament subscales	Factor 1 (PS)	Factor 2 (HA)	Factor 3 (RD)	Factor 4 (NS)	Subscale congruence
NS1	0.02	**−0.43**	0.39	0.23	0.86^*^
NS2	−0.27	−0.05	0.03	**0.71**	0.99^**^
NS3	0.02	0.11	0.30	**0.73**	0.98^**^
NS4	−0.03	−0.08	−0.20	**0.66**	0.99^**^
HA1	−0.09	**0.76**	−0.12	−0.05	0.94^**^
HA2	−0.04	**0.78**	0.20	−0.14	0.98^**^
HA3	−0.11	**0.67**	−0.27	−0.02	0.98^**^
HA4	−0.25	**0.74**	0.05	0.11	0.98^**^
RD1	0.35	**0.57**	**0.57**	0.12	0.99^**^
RD2	0.15	−0.09	**0.82**	0.18	0.98^**^
RD3	−0.16	−0.23	**0.80**	0.17	0.99^**^
RD4	−0.30	−0.06	**0.65**	**−0.49**	0.99^**^
PS1	**0.76**	−0.28	0.03	−0.05	0.97^**^
PS2	**0.85**	−0.07	−0.12	0.05	0.99^**^
PS3	**0.91**	0.05	−0.06	−0.08	0.98^**^
PS4	**0.78**	−0.04	0.16	−0.06	0.95^**^
Factor congruence	0.98^**^	0.98^**^	0.97^**^	0.94^**^	0.97^**^

Character subscales	Factor 1 (CO)	Factor 2 (SD)	Factor 3 (ST)	Subscale congruence
SD1	0.14	**0.65**	−0.32	0.98^**^
SD2	−0.18	**0.78**	0.09	0.98^**^
SD3	−0.24	**0.88**	0.08	1.00^**^
SD4	**0.69**	−0.01	−0.35	1.00^**^
SD5	0.22	**0.59**	0.04	0.94^**^
CO1	**0.51**	0.30	0.21	1.00^**^
CO2	**0.49**	0.23	0.37	0.93^*^
CO3	**0.74**	0.16	0.10	1.00^**^
CO4	**0.75**	0.03	0.11	0.99^**^
CO5	**0.85**	−0.09	0.07	0.99^**^
ST1	−0.29	0.01	**0.85**	1.00^**^
ST2	0.07	−0.00	**0.85**	0.99^**^
ST3	0.24	−0.25	**0.59**	0.97^**^
Factor congruence	0.97^**^	0.99^**^	0.99^**^	0.98^**^

**Notes.**

* Congruence ≥95% of rotations from random data.

** Congruence ≥99% of rotations from random data.

NS Novelty Seeking HA Harm Avoidance RD Reward Dependence PS Persistence SD Self-Directedness CO Cooperativeness ST Self-Transcendence

*Confirmatory factor analysis (CFA)*: When the temperament subscales were subjected to CFA of its hypothesized four-factor structure, a poor fit was observed: *χ*^2^ = 889.98; *p* < 0.001, *χ*^2^∕*df* = 9.*o*8, CFI = 0.78, GFI = 0.82, RMSEA = 0.12, SRMR = 0.12. CFA indicated that the hypothesized three-factor model of character subscales also provided a poor fit for the data: *χ*^2^ = 446.93; *p* < 0.001, *χ*^2^∕*df* = 7.21, CFI = 0.82, GFI = 0.86, RMSEA = 0.11, and SRMR = 0.08.

### Descriptive statistics, internal consistency and gender differences

Means, standard deviations and Cronbach’s alpha coefficients for the higher-oder scales and subscales are presented in Table 3. For the temperament scales, Cronbach’s alpha coefficients ranged between 0.80 (RD) and 0.93 (PS) and for the character scales between 0.87 (ST) and 0.88 (SD and CO), indicating high internal consistency. For the temperament subscales, Cronbach’s alpha coefficients ranged between 0.49 (NS4) and 0.83 (HA3) and for the character subscales between 0.57 (CO3) to 0.87 (CO4). Univariate analyses of variance revealed that women had higher HA, RD and CO scores (all *p* values <0.005) compared with men (Table 3).

**Table 3:** Descriptive statistics, internal consistency reliabilities and gender differences of the TCI-R measures. *P* values <0.05 are marked in bold.

Higher-order scales and subscales	Number of items	Cronbach’s *α*	Women (*n*=304)		Men (*n*=179)		*p* value	Cohen’s *d*
			Mean	SD	Mean	SD		
Exploratory excitability (NS1)	10	0.59	32.23	4.65	31.97	4.65	0.547	0.056
Impulsiveness (NS2)	9	0.72	24.53	4.66	23.94	5.49	0.225	0.116
Extravagance (NS3)	9	0.77	29.20	5.64	28.04	5.54	**0.029**	0.208
Disorderliness (NS4)	7	0.49	19.65	3.64	20.34	3.80	**0.048**	0.185
*Novelty Seeking (NS)*	35	0.80	105.62	12.70	104.29	13.37	0.278	0.102
Anticipatory worry (HA1)	11	0.79	30.52	6.14	28.77	6.01	**0.002**	0.288
Fear of uncertainty (HA2)	7	0.75	24.24	4.67	21.24	5.23	**<0.001**	0.605
Shyness (HA3)	7	0.83	19.76	5.62	18.77	5.39	0.056	0.180
Fatigability (HA4)	8	0.74	22.74	4.80	20.66	4.94	**<0.001**	0.427
*Harm Avoidance (HA)*	33	0.91	97.26	16.79	89.44	17.92	**<0.001**	0.450
Sentimentality (RD1)	8	0.73	29.63	4.25	27.26	4.91	**<0.001**	0.516
Openness to warm communication (RD2)	10	0.82	35.80	6.01	34.88	6.27	0.110	0.150
Attachment (RD3)	6	0.82	20.91	4.81	19.46	5.06	**0.002**	0.294
Dependence (RD4)	6	0.58	20.15	3.29	19.01	3.47	**<0.001**	0.337
*Reward Dependence(RD)*	30	0.87	106.5	13.56	100.61	14.55	**<0.001**	0.419
Eagerness of effort (PS1)	9	0.80	31.56	5.05	31.02	5.84	0.288	0.099
Work Hardened (PS2 )	8	0.79	27.76	4.32	28.57	5.08	0.062	0.172
Ambitious (PS3)	10	0.81	35.30	5.13	36.72	5.77	**0.005**	0.260
Perfectionist (PS4)	8	0.75	26.28	4.68	26.97	4.92	0.124	0.144
*Persistence (PS)*	35	0.93	120.89	16.35	123.28	19.24	0.147	0.134
Responsibility (SD1)	8	0.78	28.90	4.99	29.50	4.91	0.203	0.121
Purposefulness (SD2)	6	0.72	21.83	3.84	22.70	3.88	**0.016**	0.225
Resourcefulness (SD3)	5	0.73	17.37	3.26	18.53	3.43	**<0.001**	0.347
Self-acceptance (SD4)	10	0.82	33.76	6.75	33.15	7.76	0.361	0.084
Enlightened second nature (SD5)	11	0.76	38.87	5.61	39.01	5.80	0.785	0.025
*Self-Directedness (SD)*	40	0.88	140.72	17.01	142.88	17.33	0.181	0.126
Social acceptance (CO1)	8	0.72	29.99	4.08	29.91	4.18	0.844	0.019
Empathy (CO2)	5	0.59	18.37	2.67	17.70	2.96	**0.011**	0.238
Helpfulness (CO3)	8	0.57	29.58	3.56	28.56	3.54	**0.002**	0.187
Compassion (CO4)	7	0.87	27.36	5.19	26.09	5.47	**0.011**	0.238
Pure-hearted conscience (CO5)	8	0.59	30.18	4.24	29.20	4.48	**0.017**	0.225
*Cooperativeness (CO)*	36	0.88	135.48	14.38	131.46	15.53	**0.004**	0.269
Self-forgetful experience (ST1)	10	0.77	26.91	6.02	27.46	6.69	0.351	0.086
Transpersonal identification (ST2)	8	0.77	21.25	5.21	21.60	5.81	0.487	0.063
Spiritual acceptance (ST3)	8	0.79	20.68	5.77	19.50	5.80	**0.030**	0.204
*Self-Transcendence (ST)*	26	0.87	68.84	13.84	68.56	14.87	0.836	0.019

**Notes.**

TCI-R Temperament and Character Inventory-Revised NS Novelty Seeking HA Harm Avoidance RD Reward Dependence PS Persistence SD Self-Directedness CO Cooperativeness ST Self-Transcendence

### Correlations among TCI-R dimensions and correlations with age

The strongest correlations were between CO and RD (Pearson’s *r* = 0.59) and between SD and HA (Pearson’s *r* = − 0.54). Moderate correlations were observed between CO and SD (Pearson’s *r* = 0.45) as well as between PS and HA (Pearson’s *r* = − 0.45) and between PS and SD (Pearson’s *r* = 0.41). The remaining correlation coefficients indicated weaker relationships (Pearson’s *r* range: −0.35 to −0.02). Age correlated negatively with NS (Pearson’s *r* = − 0.16). For a detailed description of the correlation matrix see Table 4 (upper panel).

**Table 4:** Correlations between temperament and character higher-order scales and age (upper panel) and TCI-R correlations with Schizotypal Personality Questionnaire and Cognitive Failures Questionnaire (lower panel).

	NS	HA	RD	PS	SD	CO	ST
Harm Avoidance	−0.35^**^						
Reward Dependence	0.22^**^	−0.09^*^					
Persistence	−0.04	−0.45^**^	0.14^**^				
Self-Directedness	−0.02	−0.54^**^	0.19^**^	0.41^**^			
Cooperativeness	−0.07	−0.15^**^	0.59^**^	0.28^**^	0.45^**^		
Self-Transcendence	−0.13	−0.04	0.25^**^	0.25^**^	−0.17^**^	0.28^**^	
Age	−0.16^**^	0.01	0.00	−0.01	−0.02	−0.00	0.08
*Positive and Negative Affectivity Scales*							
Positive Affectivity	0.04	−0.38^**^	0.13^**^	0.55^**^	0.37^**^	0.21^**^	0.22^**^
Negative Affectivity	−0.10^*^	0.43^**^	−0.03	−0.13^**^	−0.45^**^	−0.18^**^	0.14^**^
*Schizotypal Personality Questionnaire*							
Cognitive-Perceptual	0.01	0.15^**^	0. 07	0.15^**^	−0.31^**^	−0.01	0.54^**^
Interpersonal	−0.23^**^	0.51^**^	−0.45^**^	−0.12^**^	−0.49^**^	−0.35^**^	0.10^*^
Disorganization	0.05	0.18^**^	−0.14^**^	−0.01	−0.33^**^	−0.17^**^	0.27^**^
Total score	−0.09	0.37^**^	−0.22^**^	−0 01	−0.46^**^	−0.20^**^	0.37^**^
*Cognitive Failures Questionnaire*							
Memory	−0.08	0.26^**^	0.04	−0.07	−0.30^**^	0.04	0.24^**^
Distractibility	−0.08	0.38^**^	0.03	−0.15^**^	−0.38^**^	0.04	0.22^**^
Blunders	−0.02	0.26^**^	−0.07	−0.07	−0.36^**^	−0.05	0.21^**^
Memory for Names	−0.08	0.13^*^	−0.09	−0.09	0.01	0.02	0.00
Total score	−0.08	0.34^**^	−0.01	−0.12^*^	−0.36^**^	0.02	0.23^**^

**Notes.**

** *p* < 0.01.

* *p* < 0.05.

NS Novelty Seeking HA Harm Avoidance RD Reward Dependence PS Persistence SD Self-Directedness CO Cooperativeness ST Self-Transcendence

### External validity of TCI-R

#### Correlations between TCI-R scales with PANAS, SPQ and CFQ

The correlations between TCI-R scales, PANAS, SPQ and CFQ measures are shown in Table 4 (lower panel). Positive affectivity correlated negatively with HA (Pearson’s *r* = − 0.38) and positively with RD, PS, SD, CO and ST (Pearson’s *r* range: 0.13 for RD to 0.55 for PS). Negative affectivity correlated more strongly with HA and SD (Pearson’s *r* values: 0.43 and −0.45, respectively), while only weak correlations were found with NS, PS, CO and ST (Pearson’s r range: −0.10 for NS to −0.18 for CO).

As regards correlations with the SPQ measures, NS correlated negatively only with the Interpersonal factor (Pearson’s *r* = − 0.23); HA correlated positively with all measures (Pearson’s *r* range: 0.15 for Cognitive-perceptual to 0.51 for Interpersonal factors); RD correlated negatively with all measures except the Cognitive-Perceptual factor (Pearson’s *r* range: −0.14 for Disorganization to −0.45 for Interpersonal factors); PS correlated positively with the Cognitive-Perceptual and negatively with the Interpersonal factors (Pearson’s *r* = 0.15 and −0.12, respectively); SD correlated negatively with all measures (Pearson’s *r* range: −0.31 for Cognitive-perceptual to −0.49 for Interpersonal factors); CO correlated negatively with all measures except the Cognitive-Perceptual factor (Pearson’s *r* range: −0.17 for Disorganization to −0.35 for Interpersonal factors) and ST correlated positively with all measures (Pearson’s *r* range: 0.10 for Interpersonal to 0.54 for Cognitive-Perceptual factors).

Correlations with the CFQ measures revealed that HA correlated positively with all measures (Pearson’s *r* range: 0.13 for Memory for Names to 0.38 for Distractibility). PS correlated negatively with Distractibility and Total score (Pearson’s *r* = − 0.15 and −0.12, respectively). SD also correlated negatively with all measures except Memory for Names (Pearson’s *r* range: −0.30 for Memory to −0.38 for Distractibility). ST correlated positively with all measures except Memory for Names (Pearson’s *r* range: 0.21 for Blunders to 0.24 for Memory).

#### Prediction of SPQ total score by TCI-R scales

Stepwise regression analysis with SPQ total score as the dependent variable and the Temperament scales as the predictors revealed that high NS, HA and PS along with low RD predicted high SPQ total score (*F*(4, 481): 36.6, *p* < 0.001, *R*^2^: 23.4%). Identical analysis with the Character scales as the predictors showed that low SD and CO along with high ST predicted high SPQ total score (*F*(3, 482): 75.9, *p* < 0.001, *R*^2^: 32.2%). When both the Temperament and the Character scales were included in the predictors, a similar pattern was revealed (i.e., high NS, HA, PS and ST along with low RD and SD predicted high total SPQ score but this time CO was no longer a significant predictor) and a higher percentage of the variance was explained (*F*(6, 481): 61.3, *p* < 0.001, *R*^2^: 43.6%). Including all Temperament and Character subscales as predictors in the model resulted in 48.3% explained variance (*F*(9, 481): 49.1, *p* < 0.001). For a detailed description of the significant findings in the stepwise regressions, see Table 5.

**Table 5:** Association of Temperament and Character traits with SPQ total score.

	Standardized Beta	*t*-value	*P* value
**Predictors** *(Temperament scales)*			
Novelty Seeking	0.16	3.58	<0.001
Harm Avoidance	0.53	10.71	<0.001
Reward Dependence	−0.25	−5.88	<0.001
Persistence	0.27	5.78	<0.001
**Predictors** *(Character scales)*			
Self-directedness	−0.33	−7.36	<0.001
Cooperativeness	−0.16	−3.36	0.001
Self-transcendence	0.36	8.71	<0.001
**Predictors** *(Temperament & Character scales)*			
Novelty Seeking	0.11	2.79	0.006
Harm Avoidance	0.37	7.51	<0.001
Reward Dependence	−0.29	−7.54	<0.001
Persistence	0.21	4.79	<0.001
Self-directedness	−0.23	−4.73	<0.001
Self-transcendence	0.38	9.53	<0.001
**Predictors** *(Temperament & Character subscales)*			
Shyness with strangers (HA3)	0.22	5.16	<0.001
Fatigability & asthenia (HA4)	0.15	3.48	0.001
Sentimentality (RD1)	0.14	3.14	0.002
Attachment (RD3)	−0.21	−5.27	<0.001
Work-hardened vs spoiled (PS2)	0.14	3.35	0.001
Responsibility vs Blaming (SD1)	−0.18	−4.33	<0.001
Empathy vs Social disinterest (CO2)	−0.09	−2.22	<0.03
Pure-hearted Conscience vs Self-serving Advantage (CO5)	−0.11	−2.93	0.004
Self-forgetful vs Self-conscious Experience (ST1)	0.33	8.79	<0.001

**Notes.**

HA Harm Avoidance RD Reward Dependence PS Persistence SD Self-Directedness CO Cooperativeness ST Self-Transcendence

### Personality profile analyses

The personality profiles were formulated with median splits of the total sample on every Temperament and Character dimension (for a detailed description of the personality profiles, see Table 6). The configurations are designated by capitalized letters for those above the median and lower case letters for those below the median for each dimension: Harm Avoidance (H or h), Novelty Seeking (N or n), Reward Dependence (R or r), Persistence (P or p), Self-directedness (S or s), Cooperativeness (C or c), and Self-transcendence (T or t). When there were significant between-group differences in age or years of education, these variables were included as covariates in the univariate ANOVAs examining differences in PANAS, SPQ and CFQ. Similarly, when the groups differed in gender, this variable was included as an additional grouping factor in the univariate ANOVAs. Means, standard deviations and Cohen’s d values for all the group-wise comparisons as well as the percentages of participants in each personality profile are presented in Table 7.

**Table 6:** Description of the personality profiles. Capital letters in the personality profiles indicate scores above the median and lower-case letters indicate scores below the median (e.g., NHr, high Novelty Seeking, high Harm Avoidance, low Reward Dependence).

	Descriptors & Profiles
**Temperament profiles**	Explosive (NHr) vs Reliable (nhR)
	Adventurous (Nhr) vs Cautious (nHR)
	Sensitive (NHR) vs Independent (nhr)
	Methodical (nHr) vs Passionate (NhR)
**Character profiles**	Schizotypal/Disorganized (scT) vs Organized (SCt)
	Apathetic (sct) vs Creative (SCT)
	Moody (sCT) vs Bossy (Sct)
	Fanatical (ScT) vs Dependent (sCt)
**Resilience profiles**	Fragile (Hps) vs Resilient (hPS)
	High-strung (HpS) vs Happy-go-lucky (hPs)
	Laid-back (hps) vs Conscientious (HPS)
	Perfectionist (HPs) vs Self-reliant (hpS)

**Notes.**

N Novelty Seeking H Harm Avoidance R Reward Dependence S Self-Directedness C Cooperativeness T Self-Transcendence P Persistence

**Table 7:** Descriptive statistics of the personality profile groups. Capital letters in the personality profiles indicate scores above the median and lower-case letters indicate scores below the median (e.g., NHr, high Novelty Seeking, high Harm Avoidance, low Reward Dependence). *P* values<0.05 are marked in bold.

Temperament profiles						
	Explosive (NHr; *n* = 37)	Reliable (nhR; *n* = 48)	P value (Cohen’s *d*)	Adventurous^a^ (Nhr; *n* = 51)	Cautious (nHR; *n* = 37)	P value (Cohen’s *d*)
**PANAS**						
Positive Affect	32.62 ± 4.73	37.75 ± 5.26	**<0.001 (0.776)**	35.69 ± 5.16	33.73 ± 4.82	>0.060 (0.393)
Negative Affect	23.19 ± 6.45	20.10 ± 6.63	**=0.034 (0.472)**	18.73 ± 5.74	23.14 ± 6.72	**<0.001 (0.706)**
**SPQ**						
Cognitive-Perceptual factor	10.11 ± 6.82	7.40 ± 5.44	**=0.045 (0.439)**	5.98 ± 4.87	9.24 ± 5.33	**=0.001 (0.639)**
Interpersonal factor	11.95 ± 6.32	4.65 ± 3.25	**<0.001 (1.453)**	7.27 ± 4.96	9.59 ± 5.08	**=0.001 (0.462)**
Disorganized factor	4.97 ± 3.34	2.60 ± 2.70	**=0.001 (0.780)**	3.75 ± 3.33	3.51 ± 2.48	>0.520 (0.082)
Total score	23.54 ± 12.33	12.54 ± 8.22	**<0.001 (1.050)**	14.86 ± 9.78	19.27 ± 8.98	**=0.001 (0.470)**
**CFQ**						
Memory	11.11 ± 6.23	10.09 ± 4.97	>0.480 (0.181)	7.73 ± 4.70	9.96 ± 3.80	>0.080 (0.522)
Distractibility	15.56 ± 7.46	14.27 ± 6.19	>0.460 (0.188)	11.45 ± 6.55	14.08 ± 5.14	**=0.011 (0.447)**
Blunders	10.37 ± 3.91	8.97 ± 3.58	>0.150 (0.373)	8.08 ± 4.18	7.24 ± 3.43	>0.980 (0.220)
Names	4.52 ± 2.34	4.36 ± 2.19	>0.790 (0.071)	3.95 ± 2.00	3.52 ± 1.73	>0.550 (0.230)
Total score	39.56 ± 16.88	36.12 ± 14.50	>0.400 (0.219)	30.03 ± 14.51	32.52 ± 11.16	>0.160 (0.192)
**% of total sample**	7.66 %	9.94%		10.56%	7.66%	

	Sensitive^a^ (NHR; *n* = 57)	Independent (nhr; *n* = 55)	*P* value (Cohen’s *d*)	Methodical (nHr; *n* = 88)	Passionate (nHR; *n* = 71)	*P* value (Cohen’s *d*)
**PANAS**						
Positive Affect	34.23 ± 5.01	35.69 ± 5.07	>0.900 (0.290)	33.99 ± 5.15	37.62 ± 5.47	**<0.001 (0.683)**
Negative Affect	25.21 ± 7.94	20.59 ± 6.71	**=0.003 (0.629)**	24.76 ± 5.78	19.32 ± 6.93	**<0.001 (0.853)**
**SPQ**						
Cognitive-Perceptual factor	9.44 ± 6.94	7.62 ± 5.46	>0.580 (0.291)	8.74 ± 5.65	8.73 ± 5.42	>0.780 (0.002)
Interpersonal factor	8.37 ± 5.42	8.27 ± 5.04	>0.980 (0.019)	13.27 ± 6.29	5.23 ± 3.58	**<0.001 (1.571)**
Disorganized factor	3.60 ± 2.75	2.76 ± 2.50	>0.050 (0.320)	4.35 ± 3.24	3.56 ± 3.35	>0.090 (0.240)
Total score	18.46 ± 10.68	15.64 ± 8.57	>0.380 (0.291)	22.95 ± 10.96	15.04 ± 9.56	**<0.001 (0.769)**
**CFQ**						
Memory	11.94 ± 5.88	9.79 ± 3.45	>0.620 (0.446)	11.52 ± 4.00	8.69 ± 5.21	**<0.001 (0.609)**
Distractibility	17.75 ± 6.93	12.84 ± 4.20	>0.220 (0.857)	17.59 ± 5.28	12.94 ± 6.21	**<0.001 (0.807)**
Blunders	11.50 ± 4.64	9.77 ± 2.83	>0.580 (0.450)	10.93 ± 3.80	9.33 ± 4.43	**<0.001 (0.388)**
Names	4.24 ± 2.41	3.72 ± 1.72	>0.070 (0.248)	4.59 ± 2.01	3.22 ± 2.36	**<0.001 (0.625)**
Total score	43.50 ± 16.17	34.74 ± 9.29	>0.270 (0.664)	42.92 ± 11.51	32.41 ± 14.99	**<0.001 (0.786)**
**% of total sample**	11.80%	11.39%		18.22%	14.70%	
**% of total sample that was not classified in any Temperament profile:** 8.07% (*n* = 39)						

Character profiles						
	Schizotypal/ Disorganized (scT; *n* = 85)	Organized (SCt; *n* = 76)	*P* value (Cohen’s *d*)	Apathetic (sct; *n* = 68)	Creative (SCT; *n* = 68)	*P* value (Cohen’s *d*)
**PANAS**						
Positive Affect	34.83 ± 5.18	35.68 ± 4.58	>0.270 (0.174)	31.67 ± 5.28	38.44 ± 4.63	**<0.001 (1.363)**
Negative Affect	24.39 ± 6.70	18.30 ± 4.72	**<0.001 (1.051)**	24.10 ± 6.57	19.41 ± 7.28	**<0.001 (0.676)**
**SPQ**						
Cognitive-Perceptual factor	11.84 ± 6.40	4.32 ± 3.72	**<0.001 (1.437)**	7.72 ± 5.27	10.06 ± 5.23	**=0.013 (0.446)**
Interpersonal factor	11.42 ± 5.57	5.41 ± 3.86	**<0.001 (1.254)**	12.06 ± 7.28	6.18 ± 4.10	**<0.001 (0.995)**
Disorganized factor	4.86 ± 3.49	1.89 ± 1.82	**<0.001 (1.067)**	4.29 ± 3.25	3.09 ± 3.08	**=0.028 (0.379)**
Total score	24.21 ± 11.44	9.93 ± 6.08	**<0.001 (1.559)**	20.85 ± 11.62	16.74 ± 9.37	**=0.025 (0.389)**
**CFQ**						
Memory	11.53 ± 4.44	8.61 ± 3.91	**<0.001 (0.698)**	10.65 ± 4.62	9.64 ± 4.93	>0.280 (0.211)
Distractibility	17.44 ± 6.21	12.82 ± 4.47	**<0.001 (0.854)**	15.65 ± 6.63	13.65 ± 5.55	>0.100 (0.327)
Blunders	11.21 ± 4.32	8.75 ± 3.72	**=0.002 (0.610)**	10.63 ± 3.52	8.78 ± 3.54	**=0.010 (0.524)**
Names	3.78 ± 2.11	3.94 ± 1.96	>0.720 (0.079)	4.04 ± 2.22	3.98 ± 2.26	>0.890 (0.027)
Total score	42.06 ± 13.95	32.71 ± 10.41	**<0.001 (0.760)**	39.02 ± 13.85	34.53 ± 13.45	>0.090 (0.329)
**% of total sample**	17.60%	15.73%		14.08%	14.08%	

	Moody^a^ (sCT; *n* = 53)	Bossy (Sct; *n* = 54)	*P* value (Cohen’s *d*)	Fanatical (ScT; *n* = 19)	Dependent (sCt; *n* = 25)	*P* value (Cohen’s *d*)
PANAS						
Positive Affect	34.79 ± 4.81	35.72 ± 5.02	>0.810 (0.189)	37.79 ± 4.20	33.52 ± 6.35	**=0.025 (0.793)**
Negative Affect	23.91 ± 6.37	19.93 ± 6.65	**=0.003 (0.611)**	23.11 ± 6.16	24.40 ± 7.52	>0.670 (0.188)
**SPQ**						
Cognitive-Perceptual factor	12.15 ± 6.11	4.35 ± 3.13	**<0.001 (1.607)**	10.53 ± 4.82	6.80 ± 5.37	**=0.021 (0.731)**
Interpersonal factor	9.85 ± 6.48	7.43 ± 6.21	**=0.023 (0.381)**	7.47 ± 3.81	9.52 ± 5.35	>0.230 (0.441)
Disorganized factor	4.34 ± 2.99	2.52 ± 2.20	**<0.001 (0.693)**	4.21 ± 2.51	4.00 ± 3.35	>0.910 (0.071)
Total score	22.94 ± 10.99	12.30 ± 8.58	**<0.001 (1.079)**	19.00 ± 6.62	17.56 ± 9.98	>0.520 (0.170)
**CFQ**						
Memory	12.10 ± 5.82	8.03 ± 4.96	**=0.012 (0.753)**	8.64 ± 4.27	11.23 ± 3.79	>0.210 (0.642)
Distractibility	16.93 ± 5.94	10.95 ± 6.72	**=0.002 (0.943)**	11.71 ± 4.51	16.08 ± 5.48	>0.070 (0.871)
Blunders	10.98 ± 3.72	7.00 ± 3.81	**<0.001 (1.057)**	9.43 ± 3.11	9.54 ± 3.95	>0.790 (0.031)
Names	4.00 ± 2.21	4.08 ± 2.06	>0.670 (0.037)	4.14 ± 1.83	4.15 ± 2.03	>0.630 (0.005)
Total score	42.02 ± 14.31	28.53 ± 14.49	**=0.001 (0.937)**	32.21 ± 11.43	39.62 ± 12.95	>0.260 (0.607)
**% of total sample**	10.97%	11.18%		3.93%	5.18%	
**% of total sample that was not classified in any Character profile:** 7.25% (*n* = 35)						

Resilience profiles						
	Fragile^a^^,^^b^ (Hps; *n* = 109)	Resilient (hPS; *n* = 109)	P value (Cohen’s *d*)	High-strung (HpS; *n* = 38)	Happy-go-lucky (hPs; *n* = 40)	P value (Cohen’s *d*)
**PANAS**						
Positive Affect	31.51 ± 4.51	38.79 ± 4.53	**<0.001 (1.611)**	34.57 ± 3.56	37.90 ± 4.86	**=0.001 (0.782)**
Negative Affect	25.60 ± 6.85	18.89 ± 6.50	**<0.001 (1.005)**	21.24 ± 5.22	22.98 ± 7.20	>0.230 (0.277)
**SPQ**						
Cognitive-Perceptual factor	9.83 ± 6.47	7.91 ± 5.32	>0.090 (0.324)	5.89 ± 5.25	10.25 ± 5.72	**=0.001 (0.794)**
Interpersonal factor	12.20 ± 6.73	5.71 ± 4.45	**<0.001 (1.138)**	7.89 ± 5.21	8.35 ± 3.87	>0.660 (0.100)
Disorganized factor	4.39 ± 2.94	2.67 ± 2.67	**<0.001 (0.612)**	2.76 ± 2.34	4.55 ± 3.72	**=0.014 (0.576)**
Total score	23.09 ± 11.77	13.83 ± 8.86	**<0.001 (0.889)**	14.61 ± 8.52	19.48 ± 9.51	**=0.020 (0.539)**
**CFQ**						
Memory	12.26 ± 4.37	8.76 ± 4.66	**<0.001 (0.775)**	8.86 ± 4.58	10.82 ± 4.90	>0.110 (0.413)
Distractibility	18.36 ± 5.50	12.36 ± 5.43	**<0.001 (1.098)**	12.50 ± 5.49	15.27 ± 6.40	>0.070 (0.465)
Blunders	11.20 ± 3.89	8.49 ± 3.57	**<0.001 (0.726)**	8.19 ± 4.46	11.18 ± 3.74	**=0.006 (0.726)**
Names	4.36 ± 2.01	3.98 ± 2.10	>0.160 (0.185)	4.14 ± 2.10	3.15 ± 1.91	>0.050 (0.493)
Total score	44.14 ± 12.48	32.08 ± 12.76	**<0.001 (0.956)**	32.32 ± 13.09	38.67 ± 13.85	>0.070 (0.471)
**% of total sample**	22.57%	22.57		7.87%	8.28%	


	Laid-back (hps; *n* = 37)	Conscientious (HPS; *n* = 29)	*P* value (Cohen’s *d*)	Perfectionist^b^ (HPs; *n* = 45)	Self-reliant (hpS; *n* = 46)	*P* value (Cohen’s *d*)
**PANAS**						
Positive Affect	32.17 ± 4.86	37.10 ± 4.34	**=0.001 (1.070)**	36.27 ± 4.90	34.13 ± 4.73	**=0.018 (0.444)**
Negative Affect	20.53 ± 4.84	22.21 ± 7.09	>0.110 (0.277)	25.39 ± 5.90	17.74 ± 5.79	**<0.001 (1.822)**
**SPQ**						
Cognitive-Perceptual factor	7.43 ± 4.98	8.21 ± 5.17	>0.770 (0.154)	12.62 ± 5.99	3.49 ± 3.40	**<0.001 (1.875)**
Interpersonal factor	7.08 ± 5.22	9.28 ± 4.85	>0.080 (0.437)	13.87 ± 5.80	4.57 ± 3.45	**<0.001 (1.949)**
Disorganized factor	3.95 ± 3.37	3.24 ± 2.54	>0.620 (0.238)	5.36 ± 3.70	2.20 ± 2.32	**<0.001 (1.023)**
Total score	16.38 ± 9.56	17.86 ± 8.41	>0.530 (0.164)	27.27 ± 10.39	8.91 ± 6.07	**<0.001 (2.158)**
**CFQ**						
Memory	9.50 ± 4.45	10.44 ± 4.41	>0.680 (0.212)	11.53 ± 5.66	8.10 ± 4.33	>0.060 (0.681)
Distractibility	13.83 ± 6.89	15.67 ± 5.95	>0.690 (0.286)	16.43 ± 6.20	11.17 ± 4.42	**=0.011 (0.977)**
Blunders	9.54 ± 4.24	9.78 ± 3.39	>0.710 (0.063)	10.90 ± 4.02	7.45 ± 3.50	**=0.005 (0.915)**
Names	3.46 ± 2.43	4.44 ± 2.25	>0.140 (0.418)	4.03 ± 2.37	3.62 ± 1.95	>0.820 (0.189)
Total score	34.71 ± 15.48	38.11 ± 13.34	>0.550 (0.235)	41.00 ± 14.74	29.00 ± 11.00	**=0.012 (0.923)**
**% of total sample**	7.66%	6.00%		9.32%	9.52%	
**% of total sample that was not classified in any Resilience profile:** 6.21% (*n* = 30)						

**Notes.**

a In these between-group comparisons significant gender main effects were found (all *p* values <0.05) with males scoring lower in CFQ memory (Adventurous vs Cautious and Fragile vs Resilient), distractibility (Sensitive vs Independent, Moody vs Bossy and Fragile vs Resilient) and total score (Fragile vs Resilient) compared with females.

b In these between-group comparison, significant gender main effects were found (both *p* values <0.010) with males scoring higher in PANAS Positive (Fragile vs Resilient) or PANAS Negative (Perfectionist vs Self-reliant) Affect, compared with females.

PANAS Positive and Negative Affect Schedule SPQ Schizotypal Personality Questionnaire CFQ Cognitive Failures Questionnaire N Novelty Seeking H Harm Avoidance R Reward Dependence S Self-Directedness C Cooperativeness T Self-Transcendence P Persistence

### Temperament profiles

*Explosive (NHr) vs Reliable (nhR)*. There were no between-group differences in age, years of education and gender (all *p* values >0.070). Univariate analyses of variance revealed that the “Explosive” group had lower PANAS PA and higher PANAS NA (both *p* values <0.05), SPQ Cognitive-Perceptual, Interpersonal and Disorganized factor scores, as well as higher total SPQ score (all *p* values <0.05) compared with the “Reliable” group.

*Adventurous (Nhr) vs Cautious (nHR).* The “Cautious” group was older (*p* < 0.005) and comprised of more women than men (i.e., 7 men and 30 women) compared with the “Adventurous” group (i.e., 29 men and 22 women); there were no between-group differences in years of education (*p* > 0.510). The univariate ANOVAs revealed that the “Cautious” group had higher PANAS NA, SPQ Cognitive-Perceptual, Interpersonal and total scores (all *p* values <0.001) as well as higher CFQ distractibility (*p* < 0.05) compared with the “Adventurous” group.

*Sensitive (NHR) vs Independent (nhr).* The “Sensitive” group comprised of more women than men (i.e., 9 men and 48 women) compared with the “Independent” group (i.e., 32 men and 23 women); there were no between-group differences in age and years of education (both *p* values >0.100). We found that the “Sensitive” group had higher PANAS NA (*p* < 0.005) compared with the “Independent” group.

*Methodical (nHr) vs Passionate (NhR).* The “Passionate” group was younger (*p* < 0.005) than the “Methodical” group; there were not significant in differences in years of education and gender (both *p* values >0.60). The “Methodical” group had lower PANAS PA and higher PANAS NA, SPQ Interpersonal factor score and total score (all *p* values <0.001) compared with the “Passionate” group; they also scored higher in all CFQ measures, indicating a greater subjective concern with making cognitive mistakes (all *p* values <0.05).

*Overall comparisons.* We also examined group-differences between all Temperament profiles. As the groups differed in age and education as well as gender (all *p* values <0.05), these variables served as covariates (age and education) or as additional grouping factor (gender) in the univariate ANOVAs; significant differences were followed up with Bonferroni post hoc tests.

For PANAS PA, the highest scores were obtained by the “Reliable” and “Passionate” groups who outperformed the “Explosive,” “Cautious” and “Methodical” groups (all *p* values <0.01). For PANAS NA, the highest scores were obtained by the “Sensitive” and “Methodical” groups who differed from the “Reliable,” “Adventurous”, “Independent” and “Passionate” groups (all *p* values <0.01).

As regards the SPQ, (a) the “Explosive” group tended to score higher than the “Adventurous” group in the Cognitive-Perceptual factor score (*p* = 0.056), (b) the “Explosive” and “Methodical” groups obtained the highest scores in the Interpersonal factor and differed significantly from the “Reliable,” “Adventurous” and “Passionate” groups (all *p* values <0.01), while the “Methodical” group also differed from the “Independent” and “Sensitive” groups (all p values <0.001); the “Reliable” group also scored lower compared with the “Cautious” and Independent groups (all *p* values <0.01), (c) the “Methodical” group scored higher than the “Reliable” group (*p* < 0.05) in the Disorganized factor and (d) the highest total SPQ scores were obtained by the “Explosive” and “Methodical” groups who differed from the “Reliable,” “Adventurous,” and “Passionate” groups (all *p* values <0.01) while the “Methodical” group also differed from the “Independent” group (*p* < 0.005).

Finally, in the measures of the CFQ, the “Methodical” group scored higher compared with (a) the “Adventurous” and “Passionate” groups (both *p* values <0.01) in memory, (b) the “Adventurous,” “Independent” and “Passionate” groups (all *p* values <0.01) in distractibility, (c) the “Adventurous” and “Cautious” groups (both p values <0.01) in blunders, (d) the “Passionate” group (*p* < 0.01) in memory for names and (e) the “Adventurous,” “Cautious” and “Passionate” groups (all *p* values <0.05) in the total score.

#### Character profiles

*Schizotypal/Disorganized (scT) vs Organized (SCt).* The two groups did not differ in age and gender (both *p* values >0.350) but the “Organized” group had more years of education (*p* = 0.001). We found that the “Schizotypal/Disorganized” group had higher PANAS NA, SPQ Cognitive-Perceptual, Interpersonal and Disorganized factor scores as well as higher total SPQ score compared with the “Reliable” group (all *p* values <0.001). They also reported higher CFQ memory, distractibility, blunders and total scores (all *p* values <0.005).

*Apathetic (sct) vs Creative (SCT).* There were not any significant between-group differences in all demographic variables (all *p* values >0.700). The “Apathetic” group scored lower in PANAS PA and SPQ Cognitive-Perceptual factor (both *p* values <0.05); they also scored higher in PANAS NA, SPQ Interpersonal and Disorganized factors and in SPQ total score as well as in CFQ blunders (all *p* values <0.05).

*Moody (sCT) vs Bossy (Sct).* The two groups did not differ in age (*p* > 0.460) but the “Moody” group had lower education (*p* < 0.050) and included more women than men (i.e., “Moody”: 14 men and 39 women; “Bossy”: 31 men and 23 women) compared with the “Bossy” group. We found that the “Moody” group scored higher in PANAS NA, in all measures of the SPQ and in CFQ memory, distractibility, blunders and total score (all *p* values <0.05).

*Fanatical (ScT) vs Dependent (sCt).* The “Dependent” group was younger (*p* < 0.05) but there were not between group differences in years of education and gender (both *p* values >0.090). The “Fanatical” group had higher PANAS PA and SPQ Cognitive-Perceptual factor scores (both *p* values <0.05).

*Overall comparisons.* We also examined group-differences between all Character profiles. As the groups differed in years of education and gender (all *p* values <0.05), years of education served as covariate and gender was included as additional grouping factor in the univariate ANOVAs; significant differences were followed up with Bonferroni post hoc tests.

In PANAS PA, the highest score was obtained by the “Creative” group who scored higher than the “Schizotypal,” “Apathetic” and “Bossy” groups (all *p* values <0.05) and the lowest score was obtained by the “Apathetic” group who also differed significantly from the “Schizotypal,” “Organized,” “Moody,” “Bossy” and “Fanatical” groups (all *p* values <0.01). In PANAS NA, the “Schizotypal” and “Apathetic” groups had the highest scores and differed from the “Organized”, “Creative” and “Bossy” groups (all *p* values <0.05) while the “Organized” group had the lowest score and also differed from the “Moody” and “Dependent” groups (all *p* values <0.05); the “Creative” group scored lower compared with the “Moody” group (*p* < 0.05).

As regards the SPQ, (a) the highest scores in the Cognitive-Perceptual factor were obtained by the “Schizotypal” and “Moody” groups who differed from the “Organized,” “Apathetic,” “Bossy” and “Dependent” groups (all *p* values <0.01) and the lowest scores were obtained by the “Organized” and “Bossy” groups who additionally differed from the “Creative” and “Fanatical” groups (all *p* values <0.001); (b) the highest scores in the Interpersonal factor were obtained by the “Schizotypal” and “Apathetic” groups who differed from the “Organized,” “Creative” and “Bossy” groups (all *p* values <0.05); the lowest scores were obtained by the “Organized” followed by the “Creative” groups who also differed from the “Moody” group (both *p* values <0.005); (c) the highest score in the Disorganized factor was obtained by the “Schizotypal” group followed by the “Moody” and “Apathetic” groups and they all differed from the “Organized” and “Bossy” groups (all *p* values <0.05); the “Schizotypal” group also scored higher compared with the “Creative” group and the “Fanatical” group scored higher compared with the “Organized” group (all *p* values <0.05); (d) the highest scores in the total SPQ score were obtained by the “Schizotypal” and “Moody” groups who differed from the “Organized,” “Creative” and “Bossy” groups (all *p* values <0.05) and the lowest scores were obtained by the “Organized” followed by the “Bossy” groups who additionally differed from the “Apathetic” group (all *p* values <0.001); the “Organized” group also differed from the “Creative” and “Fanatical” groups (all p values <0.05).

Finally, as regards the CFQ, significant differences were found for memory, distractibility, blunders and total score (all *p* values <0.005). In memory and distractibility, the highest scores were obtained by the “Schizotypal” and “Moody” groups who differed from the “Organized” (in memory) and from the “Organized” and “Bossy” (in distractibility) groups (all *p* values <0.05). In blunders and CFQ total score, the highest scores were obtained by the “Schizotypal” and “Moody” groups followed by the “Apathetic” group; all three groups differed from the “Bossy” group in blunders and total score (all *p* values <0.05), only the “Schizotypal” and “Moody” groups differed from the “Organized” group in blunders and total score (all *p* values <0.05) while the “Schizotypal” group also scored higher compared with the “Creative” group in blunders (*p* < 0.05).

### Resilience Profiles

*Resilient (hPS) vs Fragile (Hps).* The two groups did not differ in age and years of education (both *p* values >0.210) but there was a significant gender difference (*p* = 0.001), with the “Fragile” group consisting of more women than men (i.e., 31 men and 78 women) compared with the “Resilient” group (i.e., 54 men and 55 women). The “Fragile” group had lower PANAS PA and higher PANAS NA, SPQ Interpersonal and Disorganized as well as higher total SPQ scores compared with the “Resilient” group (all *p* values <0.001). They also reported higher CFQ memory, distractibility, blunders and total scores (all *p* values <0.001).

*High-strung (HpS) vs Happy-go-lucky (hPs).* There were no between-group differences in any demographic variables (all *p* values >0.150). The univariate ANOVAs revealed that the “High-strung” group had lower PANAS PA, SPQ Cognitive-Perceptual and Disorganized factor scores as well as SPQ total score and lower CFQ blunders score (all *p* values <0.05) compared with the “Happy-go-lucky” group.

*Laid-back (hps) vs Conscientious (HPS).* The two groups did not differ in age and years of education (both *p* values >0.230) but the “Conscientious” group consisted of more women than men (i.e., 4 men and 25 women; *p* < 0.05) compared with the “Laid-back” group (i.e., 14 men and 23 women). We found that the “Laid-back” group had lower PANAS PA (*p* = 0.001) compared with the “Conscientious” group.

*Perfectionist (HPs) vs Self-reliant (hpS).* The two groups did not differ in age (*p* > 0.690) but the “Perfectionist” group had lower education (*p* = 0.001) and comprised more women than men (i.e., 12 men and 33 women; *p* < 0.05) compared with the “Self-reliant” group (i.e., 24 men and 22 women). The univariate ANOVAs revealed that the “Perfectionist” group had higher PANAS PA and NA (both values <0.05), higher scores in all SPQ measures (all *p* values <0.001) and higher CFQ distractibility, blunders and total scores (all *p* values <0.05).

*Overall comparisons.* We also examined group-differences between all Resilience profiles. As the groups differed in years of education and gender (all *p* values <0.05), years of education served as covariate and gender was included as additional grouping factor in the univariate ANOVAs; significant differences were followed up with Bonferroni post hoc tests.

In PANAS PA, the “Resilient,” “Happy-go-lucky,” “Conscientious” and “Perfectionist” groups scored higher compared with the “Fragile” and “Laid-back” groups (all *p* values <0.05), the “Resilient” and “Happy-go-lucky” also scored higher compared with the “Self-reliant” group (all *p* values <0.005) while the “Resilient” group also differed from the “High-strung” group (*p* < 0.001). In PANAS NA, the “Fragile,” “Perfectionist” and “Happy-go-lucky” groups scored higher compared with the “Resilient” and “Self-reliant” groups (all *p* values <0.05) while the “Fragile” and “Perfectionist” groups also scored higher compared with the “Laid-back” group (all *p* values <0.05).

As regards the SPQ, (a) the highest score in the Cognitive-Perceptual factor was obtained by the “Perfectionist” group who differed from the “Fragile,” “Resilient,” “High-strung,” “Laid-back” and “Self-reliant” groups (all *p* values <0.05) and the lowest scores were obtained by the “Self-reliant” group who additionally differed from the “Fragile,” “Resilient” and “Happy-go-lucky” groups (all *p* values <0.001); the “Happy-go-lucky” group scored higher than the “High-strung” group (*p* < 0.05); (b) the highest scores in the Interpersonal factor were obtained by the “Perfectionist” and “Fragile” groups who differed from the “Resilient,” High-strung,” “Happy-go-lucky,” “Laid-back” and “Self-reliant” groups (all *p* values <0.005). The “Self-reliant” group had the lowest score and additionally differed from the “Conscientious” group (*p* < 0.05); (c) The highest score in the Disorganized factor was obtained by the “Perfectionist” group who differed from the “Resilient,” “High-strung” and “Self-reliant” groups (all *p* values <0.001). The “Self-reliant” group had the lowest score followed by the “Resilient” group and they both differed from the “Fragile” and “Happy-go-lucky” groups (all *p* values <0.05); and (d) in the SPQ total score, the highest scores were obtained by the “Perfectionist” and “Fragile” groups who differed from the “Resilient,” “High-strung,” “Laid-back” and “Self-reliant” groups (all *p* values <0.005); the “Perfectionist” group also scored higher than the “Happy-go-lucky” group (*p* < 0.005). The lowest score was obtained by the “Self-reliant” group who additionally differed from the “Happy-go-lucky” group (*p* < 0.001).

Finally, as regards the CFQ, significant differences were found for memory, distractibility, blunders and total score (all *p* values <0.001). In memory and distractibility, the “Fragile” group scored higher than the “Resilient” and “Self-reliant” groups while in distractibility only, the “Fragile” group scored higher compared with the “High-strung” and the “Laid-back” groups (all *p* values <0.05) as well. In blunders, the highest scores were obtained by the “Fragile,” ”Happy-go-lucky” and “Perfectionist” groups; all three groups scored higher than the “Self-reliant” group; the “Fragile” and “High-strung” groups also scored higher than the “Resilient” group and the “Fragile” group scored higher compared with the “High-strung” group as well (all *p* values <0.05). In total CFQ score, the “Fragile” group scored higher than the “Resilient,” ”High-strung” and “Self-reliant” groups (all *p* values <0.01).

## Discussion

In the present study we examined the psychometric properties of the TCI-R as well as associations between TCI-R dimensions with schizotypy and self-perceived cognitive functioning in a well-educated community sample. We found that specific dimensions and particular configurations of dimensions were strongly related to individual differences in affect, schizotypy, and cognitive lapses. As predicted, the “Borderline/Explosive temperament” (NHr), “Schizotypal/Disorganized character” (scT), and “Low Ego Strength/Fragile” profile (Hps) were each strongly associated with higher schizotypy, negative affect balance (low positive affect and high negative affect), and subjective cognitive lapses compared to their contrast groups. In addition, we found that people with the adventurous temperament (Nhr), moody character (sCT), and perfectionistic (HPs) profiles shared many, but not all, of these same problems. These results demonstrate the excellent psychometric properties and cross-cultural utility of the TCI-R, and the importance of considering multidimensional profiles in order to understand the influence of personality on affect and cognition in general and on schizotypy in particular.

### Psychometric Properties of the Greek TCI-R

Our results confirmed the factorial structure of Temperament and Character Inventory (Cloninger & Svrakic, 1997) with subscale scores loading on the expected factors. Nevertheless, NS1 (Exploratory excitability vs stoic rigidity) also loaded negatively on the HA and positively on the RD factors as in previous studies (Dzamonja-Ignjatovic et al., 2010; Goncalves & Cloninger, 2010; Hansenne, Delhez & Cloninger, 2005; Pelissolo et al., 2005; Snopek et al., 2012), RD4 (Dependence) also loaded negatively on the NS factor (as in Dzamonja-Ignjatovic et al., 2010; Fresán et al., 2011; Snopek et al., 2012) and SD4 (Self-acceptance vs Self-Striving) also loaded positively on the CO (as in Dzamonja-Ignjatovic et al., 2010; Fresán et al., 2011; Goncalves & Cloninger, 2010; Hansenne, Delhez & Cloninger, 2005; Pelissolo et al., 2005) and ST factors. These findings are in accordance with the existing literature in other cultural settings and further extend evidence for the complex relations between temperament and character dimensions, involving “equifinality” and “multifinality” (Cloninger, Svrakic & Svrakic, 1997). In other words, multiple temperament dimensions may be associated with the same character dimension (equifinality) and one temperament dimension may be associated with multiple character dimensions. Cloninger argues that naturally occurring dimensions of personality involve complex adaptive processes, so he used factor analytic methods only to describe the architecture of his psychobiologically based constructs, not to force them into a simple linear structure (Cloninger, 2004). The pattern of complex non-linear relations is also shown by the correlations of the higher-order scales found in the present study (for example, HA correlated with RD and PS and SD correlated with CO and ST).

The CFAs of the temperament and character subscales revealed poor indices, though. This is not a new finding, as there are similar reports with other influential omnibus personality scales in the literature (e.g., Borkeneau & Ostendorf, 1990; Church & Burke, 1994; McCrae et al., 1996). Although CFA is supposed to be “confirmatory” (i.e., the associations between the observed measurement and the primary factors are pre-determined based on the strong theoretical background of the instrument Byrne, 2005), it seems to be more suitable for simple structure models (Church & Burke, 1994) and significant limitations of this statistical approach in personality research (e.g., subscales load on multiple factors apart from the expected) have been highlighted (for a review see Hopwood & Donnellan, 2010). The “Procrustes” rotation method is another approach used to test the replicability of more complex factor structures: according to this approach, the hypothesized structures may be derived either from theory or from previous empirical results (McCrae et al., 1996). In support of the cross-cultural replicability of the TCI-R factor structure, this analysis revealed the expected mapping of the temperament and character facets onto the respective higher-order dimensions with highly significant congruence coefficients.

The internal consistency of the Greek TCI-R was high for all higher-order dimensions (Cronbach’s alpha coefficients above 0.80) and for the majority of the sub-scales (Cronbach’s alpha coefficients above 0.70) with the exception of two NS (NS1: Exploratory excitability vs stoic rigidity and NS4: Disorderliness vs Regimentation), one RD (RD4: Dependence) and two CO (CO3: Helpfulness vs unhelpfulness and CO5: Pure-hearted Conscience vs Self-serving Advantage) subscales. This was also found in the study by Farmer & Goldberg (2008) with the TCI-R. Moderate Cronbach’s alpha coefficients have also been observed with other personality measures (e.g., the Revised NEO Personality Inventory (NEO-PI-R; McCrae, Costa & Martin, 2005), the Temperament Evaluation of the Memphis, Pisa, Paris and San Diego—Autoquestionnaire (TEMPS-A; Rózsa et al., 2008). Although high internal consistency has been classically considered to be a valid index of reliability, it does not necessarily capture the multidimensionality of a scale (Schmitt, 1996; Streiner, 2003), as may be the case with the five sub-scales of the present study and personality scales in general. The gender differences reported here (i.e., women scoring higher in HA, RD and CO compared with men) are in accordance with previous studies (Fresán et al., 2011; Goncalves & Cloninger, 2010; Hansenne, Delhez & Cloninger, 2005; Pelissolo et al., 2005; Snopek et al., 2012) as is the negative correlation between age and NS (Goncalves & Cloninger, 2010; Pelissolo et al., 2005). Decreases in NS with increasing age have been observed in other studies (Fresán et al., 2011; Josefsson et al., 2013; Trouillet & Gana, 2008) possibly reflecting “*the psychological maturation of social behaviours connected with avoiding frustration and responses to novelty, impulsiveness and extravagance*” (Trouillet & Gana, 2008, p. 272).

### Associations of TCI-R dimensions with positive/negative affect and cognitive functioning

Positive and negative affectivity were assessed with the PANAS (Watson, Clark & Tellegen, 1988). Positive affectivity correlated negatively with HA and positively with RD, PS and all Character dimensions, while the opposite pattern of correlations was revealed for negative affectivity. So far, there are only two studies examining the association between Temperament and Character with positive/negative emotions as assessed with the PANAS: Cloninger & Zohar (2011) found that people with high ST or high SD also present with higher positive affect, while high SD was also significantly associated with low negative affect. Garcia, Nima & Archer (2013) reported that high positive affect is associated with high PS and low HA and that negative affect is associated with high HA and low SD in an adolescent and young adult sample. Thus, the present findings are in accordance and further extend the existing literature by revealing that CO, RD and to a lesser extent NS, are also associated with positive and/or negative affect. They also further add to the literature implicating Temperament/Character traits in disorders with dysregulated affect such as depressive and anxiety disorders (for review see Mochcovitch, Nardi & Cardoso, 2012).

Self-perceived cognitive functioning was assessed with the CFQ, a self-report questionnaire measuring the frequency of cognitive failures in everyday life (Broadbent et al., 1982). We found that high HA and high ST correlated with high self-perceived cognitive lapses, while high PS and high SD correlated with lower self-perceived cognitive lapses. This finding is in accordance and further extends studies examining the relationships between Temperament and Character dimensions and cognitive functioning (see Introduction), as examined with standard neuropsychological tasks. Also, these Temperament and Character dimensions have been associated with variations in brain structure in the frontal, temporal and parietal lobes (Gardini, Cloninger & Venneri, 2009; Kaasinen et al., 2005; Van Schuerbeek et al., 2011), brain regions that have also been associated with the severity/frequency of reported cognitive failures in everyday life (Kanai et al., 2011; Ornstein, Sahakian & McKenna, 2008); thus, we could speculate that the associations found in the present study are mediated by a neural network implicating the aforementioned regions. We could also hypothesize that high PS and SD contribute to a “more adaptive” personality with more compensatory resources providing someone with alternatives that help overcome self-perceived cognitive lapses.

### Associations between TCI-R dimensions with schizotypy

Schizotypal personality traits are traditionally assessed via interviews (e.g., the Structured Interview for Schizotypy (Kendler, Lieberman & Walsh, 1989)) or self-report scales (e.g., the Chapman Scales (Chapman, Chapman & Raulin, 1976), the Schizotypal Traits Questionnaire (Claridge & Broks, 1984), or the Schizotypal Personality Questionnaire (Raine, 1991)). The Temperament/Character personality profile characterised by increased HA and ST along with low SD and CO has been associated with schizotypy, as these traits correlated with schizotypal scores (i.e., SPQ total and factor scores correlated positively with ST and negatively with SD and CO (Daneluzzo, Stratta & Rossi, 2005) and were found to be significantly different between first-degree relatives of schizophrenia patients with high schizotypal traits and controls (i.e., first-degree relatives presented with increased HA and ST and decreased SD and CO (Bora & Veznedaroglu, 2007; Smith et al., 2008)). Confirming these findings, in the present study we found positive correlations between HA and ST with SPQ total and factor scores as well as negative correlations between SD and CO with SPQ total and factor scores. We also found a negative correlation between NS and RD with the Interpersonal factor of the SPQ as was reported in Daneluzzo, Stratta & Rossi (2005). Furthermore in the present study PS correlated negatively with the Interpersonal factor, and RD correlated negatively with the Disorganisation factor and SPQ total score.

The relationship between schizotypal traits and the TCI dimensions was further confirmed and extended with regression analyses in this study. We replicated the aforementioned relationship by finding that high HA and ST along with low SD predicted high SPQ total score and supplemented it by finding that high NS and PS and low RD are also significant predictors. Importantly, the personality profile characterized by increased HA and SD along with reduced RD, PS, SD and CO has been reported to distinguish schizophrenia patients from controls, with the largest effect sizes found for HA, SD and ST (Ohi et al., 2012). The association between ST and schizotypy is interesting due to the “dual nature” of ST: high ST is associated with high schizotypy only in combination with low SD while high ST along with high SD has been reported to indicate creativity and wisdom about life and well-being (Cloninger, 2004), suggesting developmental maturity. This pattern of associations was recently further investigated by Brambilla et al. (2014): they found the expected correlations (i.e., SD correlated negatively and ST correlated positively with schizotypy) but they also reported that both of these associations are equally explained by the same overlapping genetic and environmental factors. Low CO predicted high SPQ total score when only the Character scales were included in the predictors but with weaker loading compared with SD and ST; this possibly resulted in the exclusion of this dimension when both the Temperament and Character scales were included in the predictors.

### Personality profile differences in positive/negative affectivity, self-perceived cognitive functioning and schizotypy

When we grouped our sample according to extreme personality profiles that had been previously shown to represent non-linear dynamic systems, we found that individuals with the profiles we predicted to be most closely related to schizotypy (i.e., “Borderline/Explosive” (NHr) temperament, “Schizotypal/Disorganized” (scT) character and “Low Ego Strength/Fragile” (Hps)) profile each had lower positive and higher negative affect as well as higher schizotypal traits compared with their contrast groups (i.e., “Reliable,” “Organized” and “Resilient,” respectively). In addition, we observed similar patterns of affect and cognition in neighboring profiles (i.e., those that share two of the three components): the adventurous (Nhr) and methodical (nHr) temperaments, the moody (sCT) and apathetic (sct) characters, and the perfectionistic (HPs) and high-strung (HpS) profile had many of the same, but not all, dysfunctions in affect and cognition as those we predicted. Consequently, the multidimensional profiles capture more information than do the average effects of the individual dimensions in accounting for schizotypy, cognitive functioning, and affect balance, as has been previously observed for affect balance (Cloninger et al., 2012).

The “Borderline/Explosive” and “Schizotypal/Disorganized” profiles are highly associated with each other (Cloninger, 2004). They have both been linked with increased psychiatric morbidity (Cloninger, Bayon & Svrakic, 1998; Gurpegui et al., 2009), including depressive (Cloninger, Bayon & Svrakic, 1998; Gurpegui et al., 2009; Josefsson et al., 2011b) and anxiety symptoms (Gurpegui et al., 2009). The “Schizotypal/Disorganized” profile has also been associated with schizophrenia (Smith et al., 2008). Accordingly, the borderline, disorganized, and fragile profiles are likely to be strong early indicators of the disturbances in affect and cognition that lead to schizophrenia, which could be useful for timely recognition of individuals at risk for the schizophrenias and related disorders.

Interestingly, “Schizotypal/Disorganized,” “Methodical” and “Moody” but not “Borderline/Explosive” individuals also reported increased self-perceived cognitive failures. The “Explosive” personality is characterized by immaturity, emotional instability and strong approach-avoidance conflicts; so the association with self-perceived cognitive failures is consistent with CFQ measuring a person’s worries and doubts about his or her cognitive abilities rather than being objective performance measures of cognition per se (Wilhelm, Witthöft & Schipolowski, 2010). It is therefore possible that some subjects with these profiles may exaggerate the extent of their cognitive lapses. The schizotypal/disorganized and cyclothymic/moody characters have been shown objectively to have objective cognitive lapses often, whereas the methodical group may have an exaggerated subjective concern about cognitive lapses.

The Resilience profile bridges the Temperament and Character traits through the interaction of persistence with both Harm Avoidance and Self-directedness (Cloninger et al., 2012). In the present study, the “Low Ego Strength/Fragile” (i.e., individuals with high HA and low PS and SD) and the “Perfectionist” (i.e., individuals with high HA and PS and low SD) groups reported lower positive and higher negative affect as well as higher schizotypal traits and self-perceived cognitive failures compared with the “Resilient” and “Self-reliant” contrast groups. The neural circuitries modulating HA, PS and SD share critical regions, such as the orbitorfrontal and prefrontal cortices (Gusnard et al., 2003; Gardini, Cloninger & Venneri, 2009; Van Schuerbeek et al., 2011), which are also implicated in affective processes (Gusnard et al., 2003), schizotypy (Ettinger et al., 2012) and self-perceived cognitive functioning (Ornstein, Sahakian & McKenna, 2008). It is possible, therefore, that “Fragile” and “Perfectionist” individuals present with the aforementioned emotional, schizotypal and self-perceived cognitive profile due to alterations in this neural circuitry. However, women were over-represented in both the “Fragile” and “Perfectionist” groups, so gender effects need to be considered.

As regards the distribution of the personality profiles, we found that the “Methodical,” “Schizotypal/Disorganized” and “Fragile” configurations were the most divergent in affect, schizotypy, and cognitive lapses compared to other configurations in the Temperament, Character and Resilience networks, respectively (Table 7). The lowest prevalence was observed for the “Cautious” Temperament configuration, the “Fanatical” Character configuration and the “Conscientious” configuration in the Resilience network (Table 7). In a population-based study in Finland (Josefsson et al., 2011a) and two studies in Israel (Cloninger & Zohar, 2011; Cloninger et al., 2012), multidimensional Character and Resilience profiles as those in the present study were created. Interestingly, the pattern in the distribution of the profiles was very similar in all studies (Table 8). Taken together, these findings further support the cultural invariance in personality as suggested in Cloninger’s model and encourage further cross-cultural studies.

**Table 8:** Distribution of personality profiles in the Greek, Finnish and Israeli samples. Common rankings in the order of distribution are shaded.

	Distribution of personality profiles (highest to lowest)							
	**Temperament profiles**							
**Greek sample**	Methodical	Passionate	Sensitive	Independent	Adventurous	Reliable	Explosive	Cautious
	(18.22%)	(14.70%)	(11.80%)	(11.39%)	(10.56%)	(9.94%)	(7.66%)	(7.66%)
	**Character profiles**							
**Greek sample**	Schizotypal/Disorganized	Organized	Creative	Apathetic	Bossy	Moody	Dependent	Fanatical
	(17.60%)	(15.73%)	(14.08%)	(14.08%)	(11.18%)	(10.97%)	(5.18%)	(3.93%)
**Finnish sample** Josefsson et al. (2011a)	Schizotypal/Disorganized	Organized	Creative	Apathetic	Bossy	Moody	Dependent	Fanatical
	(19.60%)	(18.80%)	(17.80%)	(17.00%)	(8.90%)	(8.80%)	(5.00%)	(4.20%)
**Israeli sample** Cloninger & Zohar (2011)	Schizotypal/Disorganized	Organized	Creative	Apathetic	Fanatical	Dependent	Bossy	Moody
	(17.50%)	(16.80%)	(16.00%)	(15.70%)	(11.10%)	(10.70%)	(6.80%)	(5.40%)
	**Resilience profiles**							
**Greek sample**	Fragile	Resilient	Self-reliant	Perfectionist	Happy-go-lucky	High-strung	Laid-back	Conscientious
	(22.57%)	(22.57%)	(9.52%)	(9.32%)	(8.28%)	(7.87%)	(7.66%)	(6.00%)
**Israeli sample** Cloninger et al. (2012)	Fragile	Resilient	Self-reliant	Perfectionist	Happy-go-lucky	High-strung	Conscientious	Laid-back
	(24.21%)	(22.11%)	(12.98%)	(10.18%)	(8.77%)	(8.77%)	(6.67%)	(6.32%)

### Associations of gender with personality profiles

Both the “Cautious” (nHR) and (RD) and “Sensitive” (NHR) Temperament profiles included more women than men compared with the “Adventurous” and “Independent” groups, respectively, in our volunteer sample. It seems, thus, that it is the combination of high HA and RD that prevails in female volunteers irrespective of NS levels and is associated with greater need for approval and greater sensitivity to rejection, criticism, and loss (Cloninger, 2004) in accordance with the previous literature (Byrnes, Miller & Schafer, 1999; Strüber, Lück & Roth, 2008; Cross, Copping & Campbell, 2011). The “Moody” (sCT) character profile also included more women than men compared with the “Bossy” (Sct) profile, and is also associated with greater need for approval and external support. Finally, as regards the Resilience profiles, the “Fragile” (Hps), “Conscientious” (HPS), and “Perfectionist” (HPs) groups also included more women compared with their counterparts (“Resilient,” “Laid-back” and “Self-reliant,” respectively); the greatest differences was found between the “Fragile” and “Resilient” groups. In the present study we found that women have higher HA compared with men, a finding that has been well-established in previous studies even though the effect size is very small (see Miettunen et al., 2007 for a meta-analysis) and women have also been reported to score lower in PS (Goncalves & Cloninger, 2010; Gutierrez-Zotes et al., 2015) and SD (Kim, Lee & Lee, 2013). Interestingly, in the study by Kim, Lee & Lee (2013), males scored higher than females in a resilience scale. Although PS and SD correlated positively and HA correlated negatively with the resilience score in both genders, the correlations were stronger for females. It is not surprising, therefore, that the “Fragile” group (i.e., individuals with high HA and low PS and SD) was comprised of more women. We could also conclude that the “Fragile” personality profile captures even subtle limitations in capacities related to resilience in women. The associations of the “Fragile” and “Self-reliant” profiles with negative affect balance, schizotypy, and cognitive lapses indicate that these configurations have real disability, rather than representing the absence of stereotypic male characteristics.

## Conclusions

Overall, the findings of the present study extend our knowledge of the close relationship between explosive, disorganized, and fragile personality configurations with schizotypy, negative affect balance, and subjective cognitive complaints. Multidimensional profiles of temperament and character help to describe and understand the variation in the average effects of individual dimensions of personality in association with affective and cognitive processing. Our findings add support the cross-cultural applicability of the psychobiological model of personality as measured by the TCI-R, and encourage greater use of multidimensional profiles rather than considering only the average effects of its individual dimensions..

The main limitations of the study include its cross-sectional design, the fact that it was based solely on self-report measures, and that the highest percentage of the participants were females and/or had received high education, thus limiting the generalizability of the sample. Nevertheless, the sample was relatively large but had a wide age range of adults. Future studies examining the test-retest reliability of the Greek TCI-R, especially in men and in individuals with lower education compared with those in the present study, would be useful.

There is substantial information about the association of brain structure with temperament and character (e.g., Gardini, Cloninger & Venneri, 2009; Van Schuerbeek et al., 2011). In view of the strong relationships we found between the TCI and important aspects of self-reported affective, and cognitive functioning, it would be useful to extend work with the TCI by using objective neuropsychological and neuroimaging methods.

## Supplemental Information

10.7717/peerj.1830/supp-1 Data S1 Raw data

## Additional Information and Declarations

### Data Availability

The following information was supplied regarding data availability: In accordance with the funders’ requirements, raw data may only be shared upon request. Please contact Stella G. Giakoumaki (email: sgiakoumaki@uoc.gr) with your request.
